# Supplementary material for: T-Cadherin Deficiency Is Associated with Increased Blood Pressure after Physical Activity
Source: Int J Mol Sci. 2023 Sep 17;24(18):14204. doi: 10.3390/ijms241814204 (PMC10531645; doi:10.3390/ijms241814204)
Supplement: Supplementary file 1 [file ijms-24-14204-s001.zip › ijms-2575709-supplementary.pdf]

Supplementary Materials

	Prd- fam (2 exon)	Pri- hex(3 exon)
**1**	27	26,7
**2**	28,95	
**3**	32,7	
**4**	32,8	40,1
**5**	27,85	27,25
**6**	30,1	
**7**	30,35	36,5
**8**	30,05	
**9**	30,35	33,05
**10**	39,9	
**11**	31,2	37,4
**12**	30,15	
**13**	33,75	38,2
**14**	34,6	37,9
**15**	25,9	
**16**	32,7	
**17**	34,85	37,1
**18**	35,2	
**19**	33,75	
**20**	24,85	
**21**	25,1	
**22**	24,15	
**23**	23,65	
**24**	27,45	
**25**	24,25	
**26**	24,65	
**27**	23,4	
**28**	23,6	
**29**	23,55	
**30**	26,85	
**31**	24,8	
**32**	26,2	
**33**	25,05	
**34**	26,2	
**35**	26,35	
**36**	26	
**37**	26	
**38**	26,2	
**39**	23,8	
**40**	26,9	26,25
**41**	28,25	27,7
**42**	25,8	24,05

**43**	27	25,2
**44**	28,55	27
**45**	24,25	-
**46**	24,1	39
**47**	23,1	-
**48**	23,35	38,8
**49**	23,8	39,15
**50**	24,3	-
**51**	23,25	-
**52**	23,45	38,7
**53**	27,9	-
**54**	27,7	-
**55**	30,9	-
**56**	28,35	39,5
**57**	26	24,5
**58**	24,2	23
**59**	24,7	23
**60**	22,8	21,4
**61**	24,2	22,7
**62**	24	22,8
**63**	28	22,3

Table S1. Results of PCR amplification of the products corresponding to exons 2 and exon 3 in *Cdh13* gene. A total of 63 mice were examined. Amplification was detected in two channels: mice with genomic DNA being amplified in both channels (FAM and HEX) were considered to be WT, while those with amplification only in the FAM channel were considered to be T-cadherin-deficient mice lacking the 3rd exon. The *Plaur* gene, was used as a reference gene for the isolated murine genomic DNA.
